# Imported arboviral infections in Italy, July 2014-October 2015: a National Reference Laboratory report

**DOI:** 10.1186/s12879-017-2320-1

**Published:** 2017-03-16

**Authors:** Claudia Fortuna, Maria Elena Remoli, Caterina Rizzo, Eleonora Benedetti, Cristiano Fiorentini, Antonino Bella, Claudio Argentini, Francesca Farchi, Concetta Castilletti, Maria Rosaria Capobianchi, Lorenzo Zammarchi, Alessandro Bartoloni, Nadia Zanchetta, Maria Rita Gismondo, Luca Ceccherini Nelli, Giustina Vitale, Franco Baldelli, Pierlanfranco D’Agaro, Giuseppe Sodano, Giovanni Rezza, the Arbovirus Working Group, Alessia Caratelli, Veronica Bizzotti, Daniela Casale, Debora Lepore, Valentina Cecchetti, Maria Grazia Caporali, Licia Bordi, Fabrizio Carletti, Francesca Colavita, Eleonora Lalle, Serena Quartu, Lisa Malincarne, Ilaria Caracciolo, Claudia Tiberio, Erasmo Falco, Giulietta Venturi

**Affiliations:** 1 Department of Infectious Diseases, Istituto Superiore di Sanità, Rome, Italy; 2 National Center for Epidemiology and Health Promotion, Istituto Superiore di Sanità, Rome, Italy; 3 Laboratory of Virology, National Institute for Infectious Diseases Lazzaro Spallanzani, IRCCS, Rome, Italy; 4 Infectious and Tropical Diseases Unit, Careggi University Hospital, Florence, Italy; 5 Department of Experimental and Clinical Medicine, University of Florence, Florence, Italy; 6 Clinical Microbiology, Virology and Bioemergency, Luigi Sacco Hospital, Milan, Italy; 7 Virology Section and Retrovirus Centre of the Department of Translational Research NSMT, University of Pisa, Pisa University Hospital (Azienda Ospedaliero-Universitaria Pisana), Pisa, Italy; 8 Department of Laboratory Diagnosis, Palermo University Hospital (Azienda Ospedaliera Universitaria Policlinico Palermo), Palermo, Italy; 9 Clinic of Infection Diseases, Department of Medicine, University of Perugia, Perugia, Italy; 10 Department of Medical, Surgical and Health Sciences, University of Trieste, Trieste, Italy; 11 Institute for Maternal and Child Health—IRCCS Burlo Garofolo, Trieste, Italy; 12 UOC Microbiology and Virology, Hospital for Infectious Diseases “D. Cotugno”, AO dei Colli (Monaldi, Cotugno, CTO), Naples, Italy

## Abstract

**Background:**

Imported cases of infections due to Dengue (DENV) and Chikungunya (CHIKV) viruses and, more recently, Zika virus (ZIKV) are commonly reported among travelers returning from endemic regions. In areas where potentially competent vectors are present, the risk of autochthonous transmission of these vector-borne pathogens is relatively high. Laboratory surveillance is crucial to rapidly detect imported cases in order to reduce the risk of transmission. This study describes the laboratory activity performed by the National Reference Laboratory for Arboviruses (NRLA) at the Italian National Institute of Health in the period from July 2014 to October 2015.

**Methods:**

Samples from 180 patients visited/hospitalized with a suspected DENV/CHIKV/ZIKV infection were sent to the NRLA from several Italian Hospitals and from Regional Reference Laboratories for Arboviruses, in agreement with the National Plan on human surveillance of vector-borne diseases. Both serological (ELISA IgM test and Plaque Reduction Neutralization Test—PRNT) and molecular assays (Real Time PCR tests, RT-PCR plus nested PCR and sequencing of positive samples) were performed.

**Results:**

DENV infection was the most frequently diagnosed (80 confirmed/probable cases), and all four genotypes were detected. However, an increase in imported CHIKV cases (41 confirmed/probable cases) was observed, along with the detection of the first ZIKV cases (4 confirmed cases), as a consequence of the recent spread of both CHIKV and ZIKV in the Americas.

**Conclusions:**

Main diagnostic issues highlighted in our study are sensitivity limitations of molecular tests, and the importance of PRNT to confirm serological results for differential diagnosis of Arboviruses. The continuous evaluation of diagnostic strategy, and the implementation of laboratories networks involved in surveillance activities is essential to ensure correct diagnosis, and to improve the preparedness for a rapid and proper identification of viral threats.

## Background

Vector-borne viral diseases cause a substantial public health burden in tropical and sub-tropical regions. Their geographic distribution is expanding, due to many and complex factors, such as urbanization, climate change, land-use changes, human mobility, and vector range expansion [1].

The Dengue virus (DENV) is a flavivirus (family *Flaviviridae*) transmitted to humans through *Aedes (Ae.) spp* mosquito bite. Dengue fever is typically characterized by fever, myalgia, arthralgia, rash, and sometimes severe and life-threatening clinical symptoms. Dengue global incidence has increased 30-fold in the last 50 years [2], and areas with predominant circulation of a single DENV serotype have changed toward co-circulation of different virus serotypes [3]. During the past decade, additional mosquito-borne viruses, including Chikungunya virus (CHIKV) and Zika virus (ZIKV), have successfully spread to geographical areas where only dengue epidemics used to be reported [3–8]. CHIKV is an alphavirus (family *Togaviridae*) that causes an acute febrile illness characterized by severe arthralgia, whereas ZIKV, another mosquito-borne flavivirus closely related to DENV, mostly causes mild fever, joint pain, conjunctivitis, and rash [3]. As for DENV, CHIKV and ZIKV are transmitted between humans by *Ae.* species mosquitoes [9, 10]. Since 2004, CHIKV has caused epidemics in Africa, Asia, and Indian Ocean islands. In 2007 an outbreak of chikungunya originated from an imported case coming from India occurred in Italy, causing more than 200 cases of disease [11]. In December 2013, CHIKV was notified in the Caribbean and has since spread to several countries in the Americas [7, 12]. The first outbreak of ZIKV outside Africa and Asia was reported in 2007 in the Yap State, Federated States of Micronesia [13]. Subsequently, in 2013, this virus reappeared in French Polynesia and then spread throughout the Pacific. In the early 2015, the first local transmission of ZIKV was reported in Brazil [14]. Since then, the infection has rapidly spread throughout South America, Central America, and the Caribbean [8, 15, 16], and recently in Florida, USA [17]. ZIKV, previously thought to be associated with a mild clinical disease, was found to be associated with a 20-fold increase in the Guillain-Barrè syndrome incidence following the French Polynesia outbreak [18]. Moreover, the report of a possible association between ZIKV infection and an epidemic of microcephaly among neonates in Brazil has attracted global attention, and has led the World Health Organization (WHO) to declare the ZIKV epidemic as a global public health emergency on February, 1st 2016 [19]. In the meanwhile, evidence supporting the association between ZIKV infection and neonatal microcephaly and other birth defects has increased [20–25].

Imported cases of illness due to DENV and CHIKV, and more recently ZIKV, are reported every year among travelers returning from endemic regions [26–28]. In areas where competent vectors are present, the risk of autochthonous transmission of these vector-borne pathogens is particularly high [11, 29]. Thus, epidemiological and laboratory surveillance is crucial to rapidly identify imported cases in order to introduce measures to reduce risks for public health. The aim of the present study is to present data on imported infections in Italy, diagnosed at the National Reference Laboratory for Arboviruses (NRLA) in the period from July 2014 to October 2015, mainly focusing on diagnostic issues, countries of origin of the infections, and viral strains involved in the imported cases.

## Methods

### Patients and samples

Samples of patients visited/hospitalized with a suspected DENV/CHIKV/ZIKV infection, collected from July 2014 through October 2015, were analyzed. A case-report form containing information about age, sex, countries visited, travel dates, and date of onset of symptoms was completed for each patient. Samples were collected and sent to the NRLA at the Istituto Superiore di Sanità in Rome from several Italian Hospitals, in agreement with the National Plan on human surveillance of imported and autochthonous vector-borne diseases (CHIKV, DENV, ZIKV, and West Nile virus—WNV) [30, 31]. Samples were sent also from Regional Reference Laboratories for Arboviruses involved in the surveillance National Plan for diagnostic confirmation and/or with the aim of a cross-evaluation of the diagnostic methods used in different laboratories. ELISA IgM and real time PCR tests were performed for a first line diagnosis. Plaque Reduction Neutralization Tests (PRNTs) were performed to confirm positive results obtained by ELISA tests, and to discriminate between closely related viruses.

### Serological assays

#### ELISA IgM

IgM antibodies against DENV, CHIKV, and ZIKV were detected in patients serum samples using commercial IgM-capture ELISA systems (Focus Diagnostics Dengue Virus IgM Capture, DxSelect™, California, USA, NovaLisa® Chikungunya IgM μ-capture ELISA, NovaTec Immundiagnostica GmbH, Germany, Euroimmun Anti-Zika Virus IgM ELISA, Luebeck, Germany). Absorbance was measured at 450 nm using an ELISA reader, according to manufacturer’s instructions. Sample optical density readings were compared with reference cut-off OD readings to determine results. Index values >1.00 for DENV, > 11.00 for CHIKV, and > 1,1 for ZIKV were considered presumptive for the presence of IgM antibodies.

#### Plaque Reduction Neutralization test (PRNT)

The assay was performed in six-well tissue culture plates with subconfluent VERO cell monolayers (approximately 70% confluence). The following viruses were used: serotype 2 DENV (NGB strain), a CHIKV strain isolated from a patient during the 2007 Italian outbreak [11], and ZIKV H/PF/2013 strain of the Asian genotype (kindly provided by Dr. Isabelle Leparc-Goffart of the French National Reference Center on Arboviruses in Marseille) [32]. Infectivity titration of each viral strain was performed by plaque assay using VERO cells. Patients sera were diluted 1:10 in serum-free maintenance medium, heat-inactivated, and titrated in duplicate in twofold dilution steps. Equal volumes (100 μl) of DENV/CHIKV/ZIKV dilution containing approximately 80 Plaque Forming Units (PFU), and serum dilutions, were mixed, and incubated overnight at 4 °C. Subsequently, VERO cells plates were infected with 200 μl/well of virus-serum mixtures in duplicate. After 1 h incubation at 37 °C and 5% CO_2_, the inocula were aspirated and the wells were overlayed with a mixture of one part 2% Gum Tragacanth and one part of supplemented medium (2× MEM, 2.5% inactivated FCS and 2% 1 M HEPES). The plates were incubated at 37 °C and 5% CO_2_ for 2 (CHIKV) - 7 (DENV) - 4 (ZIKV) days, and then were stained with 1.5% crystal violet. A titration of CHIK/DEN/ZIK viruses with three dilutions in duplicate (the working dilution, 1:2 and 1:8 dilutions) was performed in each assay and used as a control for the assay. Neutralizing antibody titers were calculated as the reciprocal of the serum dilution that gave a 50 or 80% reduction of the number of plaques (PRNT50/PRNT80), as compared to the virus control. PRNT80 ≥ 10 were considered positive, while PRNT50 ≥ 10 were considered as border line (b.l.).

### Molecular diagnosis

#### RNA extraction and Real Time PCRs

Molecular tests were performed on acute sera of DENV/CHIKV/ZIKV-suspected patients.Viral RNA was extracted from 140 μl of serum sample by using QIAmp viral RNA Mini kit (Qiagen Inc., Valencia, CA, USA), according to the manufacturer’s instructions, and then stored at -80 °C until further processing. The RNA was amplified by real time PCR for CHIKV, DENV, and/or ZIKV detection. The primers and probes used in this study are listed in Table 1 [11, 33, 34]. All real time PCR assays were performed by using the RealTime ready RNA Virus Master mix (Roche Diagnostics, Basel, CH), according to the manufacturer’s protocol, and CFX96 Touch™ Real-Time PCR Detection System (Bio-Rad).

**Table 1:** Primers and probes for the molecular diagnosis

Primers and Probes	Sequence (5’- 3’)	Reference
DenS	GGATAGACCAGAGATCCTGCTGT	[33]
DenAs + DenAs1	CATTCCATTTTCTGGCGTTC + CAATCCATCTTGCGGCGCTC	
DenP	FAM-CAGCATCATTCCAGGCACAG-TAMRA	
ChikS	TGATCCCGACTCAACCATCCT	[11]
ChikAs	GGCAAACGCAGTGGTACTTCCT	
ChikP	FAM-TCCGACATCATCCTCCTTGCTGGC-Black Hole Quencher 1	
Zvf1086	CCGCTGCCCAACACAAG	[34]
Zvr1162c	CCACTAACGTTCTTTTGCAGACAT	
ZvP_1107	6FAM-AGCCTACCTTGACAAGCAGTCAGACACTCAA-TAMRA	

#### Amplification and sequencing from viral RNA

For DENV and CHIKV nucleic acid detection and genotyping, Reverse Transcription (RT)-PCR followed by nested PCR amplification was performed. An amplicon of 434 bp in the E gene region and an amplicon of 536 bp in the E1 structural glycoprotein coding gene region were obtained for DENV an CHIKV, respectively. The primers used for RT-PCR plus nested PCR and sequencing are listed in Table 2 [35, 36]. SuperScript One-step RT-PCR with Platinum Taq kit (Invitrogen, Gaithersburg, MD) and Platinum PCR SuperMix kit (Invitrogen) were used for RT-PCR and nested PCR, respectively. PCR products were purified by QIAquick PCR Purification Kit (Qiagen) and were sequenced on both strands by using nested forward and reverse primers.

**Table 2:** Primers for DENV and CHIKV amplification and sequencing

Primers	Sequence (5’- 3’)	Reference
RT-PCR:		[35]
DEUL	TGGCTGGTGCACAGACAATGGTT	
DEUR	GCTGTGTCACCCAGAATGGCCAT	
Nested PCR:		
DENUL	GATCTCAAGAAGGAGCCATGCA	
DENUR	ATGGAACTTCCCTTCTTGAACCA	
RT-PCR:		[36]
CHIK 10264 F	GGCGCCTACTGCTTCTG	
CHIK 11300R	CGACACGCATAGCACCSC	
Nested PCR:		
CHIK 10564 F	CCCTTTGGCGCAGGAAGAC	
CHIK 11081R	GACTTGTACGCGGAATTCGG	

### Phylogenetic analysis

The sequences obtained were aligned with other DENV/CHIKV sequences available in the GenBank database (accession numbers are reported in the phylogenetic trees), by using the ClustalW program (www.clustal.org) [37]. Alignments were manually edited with the Bioedit program [38]. Nucleotide Tamura-Nei model and the Neighbour-Joining method was used to construct the phylogenetic trees [39]. The Neighbour-Joining method was implemented by using MEGA version 6.06 (www.megasoftware.net) [40]. The robustness of branching patterns was confirmed with a bootstrap analysis using 1000 replicates.

## Results

### Dengue, Chikungunya and Zika diagnostic tests results, and areas of origin of the imported infections

Samples collected from 180 patients visited/hospitalized with a suspected DENV/CHIKV/ZIKV infection were analyzed. Of the patients, 50,6% were males, median age was 38 years (range 1–80 years). For 116 patients for whom the information about the date of symptoms onset and/or hospitalization was available, the median lag time before sample collection was 8 days (range 2–102 days, mean ± standard deviation: 15,47 ± 18,24 days). Two serum samples (acute phase and convalescence phase) were available from 27 patients. Samples were sent to the NRLA from several Italian Regions (Friuli Venezia Giulia, Lombardia, Piemonte, Liguria, Toscana, Umbria, Lazio, Abruzzo, Campania, Sardegna, Calabria e Sicilia). Most of the samples were collected during the summertime, from June to September, when the surveillance is increased because of vector activity.

On the base of diagnostic tests results, and clinical and epidemiological data, each case was defined as confirmed, probable, possible or not confirmed, according to the criteria shown in Table 3.

**Table 3:** Case definition on the bases of the diagnostic test results

Confirmed	PCR positive and/or IgM positive plus PRNT positive^a^, and/or seroconversion or four fold increase in neutralizing antibody titers in two consecutive samples.
Probable	IgM positive plus PRNT border line^b^ in acute samples^c^.
Possible	IgM negative and PRNT positive/border line, or IgM positive but PRNT negative in acute samples.
Not confirmed	IgM positive and PRNT negative in late/convalescent samples, or PRNT positive without an increase in the titer in two consecutive samples.

^a^PRNT80 ≥ 10: positive

^b^PRNT50 ≥ 10: border line (b.l.)

^c^These cases were classified as possible if PRNT b.l. results were obtained toward different viruses

In the study period, a total of 157 patients were tested for DENV, 97 for CHIKV and 16 for ZIKV (Table 4).

**Table 4:** DENV, CHIKV and ZIKV diagnosis in the period from July 2014 to October 2015

	Total	Confirmed	Probable	Possible	Not confirmed
DENV diagnosis	157	68	12	33	44
CHIKV diagnosis	97	35	6	14	42
ZIKV diagnosis	16	4^a^	0	3^b^	9
Dual diagnosis DENV/CHIKV	76: two cases of possible co-infections.	DENV: 18/76 (of which two CHIKV confirmed and one CHIKV possible cases)			
		CHIK: 20/76 (of which two DENV confirmed and 6 DENV possible cases)			

^a^The diagnosis of one of these cases was performed in Germany after we excluded DENV and CHIKV infections (ref). One was a case of autochthonous (most likely sexual) transmission (ref)

^b^Of these, two were probable cases of past ZIKV infections (PRNT positives and IgM negatives). One showed instead a PRNT b.l. result for ZIKV, which was probably due to cross reactivity of DENV specific antibodies

Overall, 68 DENV cases (plus 12 probable cases), 35 CHIKV cases (plus 6 probable cases), and 4 ZIKV cases [27, 41] were confirmed, plus two cases of ZIKV past infection. For 76 patients, diagnostic tests both for DENV and for CHIKV were performed, and 2 cases of possible co-infections were detected. Clinical features of DENV, CHIKV and ZIKV confirmed/probable cases are shown in Table 5.

**Table 5:** Clinical features of DENV, CHIKV and ZIKV confirmed/probable cases

Symptoms	DENV confirmed and probable cases presenting with the symptom^a^	CHIKV confirmed and probable cases presenting with the symptom^a^	ZIKV confirmed and probable cases presenting with the symptom^a^
Fever (≥38 °C)	93,2%	92,6%	75,0%
Arthralgia	71,2%	96,3%	75,0%
Rash	33,9%	66,7%	100,0%
Asthenia	76,3%	70,4%	25,0%
Headache	62,7%	37,0%	0,0%
Myalgia	52,6%	63,0%	25,0%
Retro-orbital pain	32,2%	11,1%	0,0%
Meningoencephalitis	1,7%	3,7%	0,0%
Others	10,2%^b^	3,7% ^c^	0,0%

^a^Symptoms were known for 59, 27 and 4 DENV, CHIKV and ZIKVV cases, respectively

^b^diarrhea, vomit, leuco-thrombocytopenia

^c^symptoms persisting for longer than 30 days

The area of origin of the suspected imported cases was known for 62 DENV, 30 CHIKV, and 4 ZIKV confirmed/probable cases DENV cases were imported from many different countries in all continents except Europe. Among DENV confirmed/probable cases, 59.7% were from Asia, 17.7% from Central and South America, 11.3% from the Caribbean, 6.5% from Africa, and 4.8% from Oceania. As expected, because of the recent CHIKV epidemics in the Americas, among the CHIKV confirmed/probable cases, 53.3% were from the Caribbean, 36.7% were from Central and South America, and only 10.0% were from Asia or Africa. Two ZIKV confirmed infections had been acquired in Brazil, one in March [27] and one in May, 2015, while one had been acquired in Thailand in 2014, and one was an autochthonous case likely due to sexual transmission [41].

### Serological diagnosis of DENV, CHIKV and ZIKV infections

#### ELISA IgM tests

Results of ELISA IgM tests are summarized in Table 6.

**Table 6:** DENV/CHIKV/ZIKV ELISA IgM tests results

	ELISA IgM: positives/tested	Estimated proportion of false positive and false negative test results
DENV (Focus Diagnostics Dengue Virus IgM Capture, DxSelect™)	55/127	8/55 (14.5%) false positives
		8/72 (11.1%) false negatives
CHIKV (NovaLisa® Chikungunya IgM μ-capture ELISA, NovaTec Immundiagnostica)	31 + 3 b.l./86	1 b.l./86 (1,2%) false positives
		5/52 (9,6%) false negatives
ZIKV (Euroimmun IgM ELISA)	3/5	

DENV ELISA IgM test was performed for 127 of 157 patients tested for DENV, with 55 positive results; for 14.5% of the ELISA IgM positive patients, a final diagnosis of confirmed or probable DENV infection was not done after considering all laboratory findings and available epidemiological data. These cases are likely to represent false positive IgM ELISA results. Conversely, 11.1% of the ELISA IgM negative patients were diagnosed as confirmed DENV cases on the basis of other tests (PCR positivity) and/or of IgM results obtained by the hospital/laboratory where the sample had been collected (not shown); they were considered as false negatives. Of note, of the DENV suspected cases coming to our laboratory with a positive IgM result obtained in the hospital/laboratory where the sample had been collected (*n* = 53) (not shown), 24.5% (13/53) could not be confirmed by PRNT nor by molecular tests.

CHIKV ELISA IgM test was performed for 86 of 97 patients tested for CHIKV, with 3 b.l. results, and 31 positive results; all positive ELISA IgM results and two of the b.l. were confirmed by a positive or b.l. PRNT result. Among 52 CHIKV ELISA IgM negative samples, 13 were positive and 4 b.l. in PRNT: of these, at least 5 (9.6%) were considered to be associated with a recently acquired infection, based on clinical and epidemiological data, and thus estimated as probable false-negative ELISA results. Of the CHIKV suspected cases coming to our laboratory with a positive IgM result obtained in the hospital/laboratory where the sample had been collected (*n* = 27) (not shown), 11.1% (3/27) were classified as not confirmed after evaluation of all laboratory findings. ZIKV IgM test was performed for 5 patients, all with a PRNT positive result for ZIKV: 3 were positive, and were thus considered as recent, confirmed, ZIKV infections, while the two IgM negatives were considered as past infections.

Overall, 94 DENV and 40 CHIKV ELISA IgM results could be compared with the IgM results obtained with different methods in the hospital/laboratory of origin of the samples (not shown): concordant results were obtained in 81.9 and 87.5% of cases for DENV and CHIKV, respectively.

#### PRNTs

PRNTs results are summarized in Table 7: neutralizing antibodies were detected in 79/157 (50.3%) of patients tested for DENV, in 47/97 (48.4%) of patients tested for CHIKV, and in 5/15 (33,3%) of patients tested for ZIKV. In 26/79 (32,9%) of DENV PRNT positive patients, 10/47 (21,2%) of CHIKV PRNT positive patients, and 2/5 (40%) of ZIKV PRNT positive patients, both molecular tests and ELISA IgM gave negative results: these subjects had probably acquired a DENV and/or CHIKV infection in the past, which was not associated with the recent/ongoing illness. A b.l. PRNT result was obtained for 31 of 157 DENV tested patients. Of these, 10 (32.3%) were classified as confirmed cases, since the viral genome could be detected in the same sample, and/or a fully positive PRNT result was obtained in a second, convalescent sample. Moreover, 12 (38.7%) were classified as probable cases, since a positive ELISA IgM results was obtained in the same sample. Finally, 9 (29%) PRNT b.l. results were obtained from cases defined as possible, which were not associated with any other positive result (*n* = 5), and/or associated with a confirmed infection by a closely related Flavivirus (ZIKV, *n* = 3), and/or in cases showing b.l. PRNT results also for different viruses (such as CHIKV and WNV, *n* = 3), suggesting a broad and non DENV-specific cross-reactivity. With respect to CHIKV diagnosis, a b.l. PRNT result was obtained for 11 (11.3%) of 97 CHIKV tested patients: one from a confirmed case, 4 from probable cases, and 6 which were classified as possible cases, since they were not associated with positive results in other CHIKV tests, and, in 3 cases, presented b.l. PRNT results also for different viruses (such as DENV and WNV). Finally, one b.l. PRNT result was obtained for ZIKV, in a sample of a DENV confirmed case.

**Table 7:** DENV/CHIKV/ZIKV PRNTs results

	PRNT positives/tested	PRNT border line/tested	
DENV	79/157 (50.3%)	31/157 (19,7%)	10/31 (32.3%): confirmed cases
			12/31 (38.7%): probable cases
			9/31 (29%): possible cases
CHIKV	47/97 (48.4%)	11/97 (11,3%)	1/11 (9%): confirmed case
			4/11 (36,4%): probable cases
			6/11 (54,6%): possible cases
ZIKV	5/15 (33,3%)	1/15 (6,7%)	possible case (probable cross reactivity of DENV specific neutralizing antibodies)

### Molecular diagnosis of DENV and CHIKV infections, and phylogenetic analysis of viral sequences

For DENV diagnosis, 25 of 132 (18.9%) samples tested by real time PCR gave a positive result. All PCR positive samples (for which the time from the onset of symptoms was known), had been collected within 8 days from the onset of symptoms (mean ± standard deviation: 4.71 ± 1.76 days). For CHIKV diagnosis, only 2/76 (2.6%) samples tested by real time PCR gave a positive result, which had been collected 3 days after the onset of symptoms. All samples analyzed for ZIKV by real time PCR gave negative results.

Among all samples collected within 8 days from the onset of symptoms, CHIKV viral genome was detected in 7.7% (2/26) of the samples (22.2% of confirmed/probable cases), and DENV viral genome in 36.8% (21/57) of tested samples (53.8% of confirmed/probable cases). These data may suggest a longer duration of viremia in DENV infection compared to CHIKV infection.

**Fig. 1:**
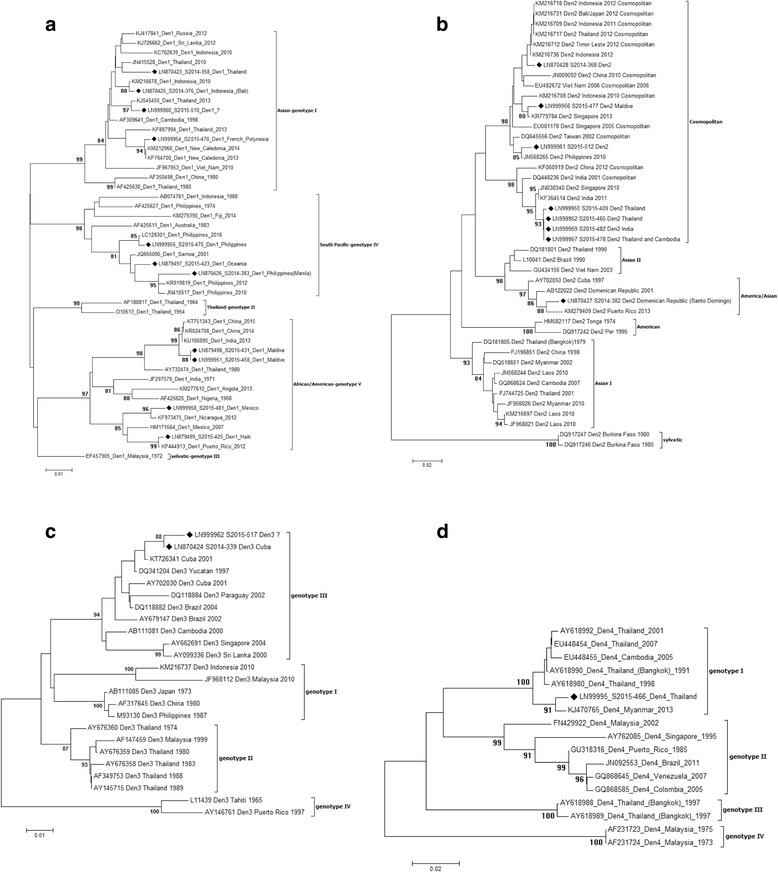
Neighbour-Joining phlylogenetic analysis of sequences obtained from DENV positive samples using Tamura-Nei model with 1000 bootstrap reiterations. For each sequence, GenBank accession number/viral genotype/country of origin of the infection/year of the infection are reported. Sequences characterized in this study are indicated by a black square. The bars indicate the percentage of diversity. Bootstrap values over 80% obtained from 1000 replicate trees are shown for key nodes. **a**: DENV-1 genotypes; **b**: DENV-2 genotypes; **c**: DENV-3 genotypes; **d**: DENV-4 genotypes

**Fig. 2:**
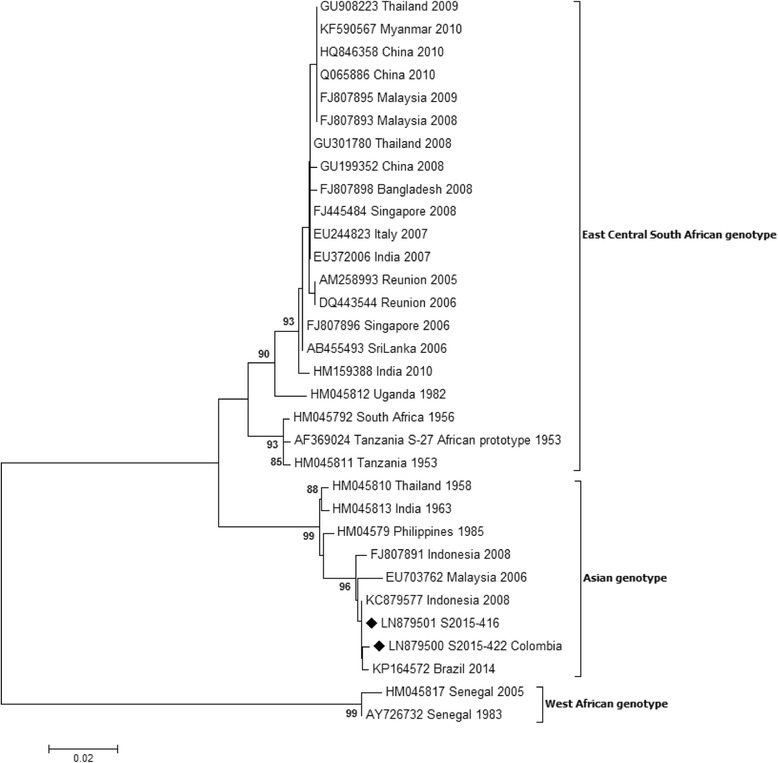
Neighbour-Joining phlylogenetic analysis of sequences obtained from CHIKV positive samples using Tamura-Nei model with 1000 bootstrap reiterations. For each sequence, GenBank accession number/viral genotype/country of origin of the infection/year of the infection are reported. Sequences characterized in this study are indicated by a black square. The bars indicate the percentage of diversity. Bootstrap values over 80% obtained from 1000 replicate trees are shown for key nodes

Sequences were obtained for 22 of the DENV PCR positive samples, and for the 2 CHIKV PCR positive samples. Both nucleic acid and translated amino acid sequences were aligned with GenBank sequences of isolates with known dates and locations, and phylogenetic analysis was performed. Viral strains and genotypes, locations of origin, year of the infections, and Gene Bank accession numbers are summarized in Tables 8 (sequences characterized in this study) and Table 9 (reference sequences). As shown in Fig. 1a,b,c and d, DENV strains of all the four serotypes were identified (11 DENV-1, 8 DENV-2, two DENV-3 and one DENV-4 strains). Within the DENV-1 serotype, 4 strains grouped together with the Asian lineage (genotype I), 3 with the South Pacific lineage (Genotype IV) and 4 with African/American lineage (Genotype V), with bootstrap values ≥ 97 (Fig. 1a). Most of DENV-1 sequences (from patients S2015-423, S2015-475, S2014-376, S2015-510, S2015-470, S2015-425, S2015-481, S2015-458, S2015-431) showed a high similarity with GenBank strains known to circulate in the areas of origin of the imported infections (88–98% homology at nucleic acid level and 100% at amino acid level). In contrast, two of our DENV-1 sequences showed a higher degree of divergence when compared to DENV-1 sequences available in Gene Bank. A strain from Philippines, identified in the patient S2014-383, showed the highest similarity (88% homology at nucleic acid level and 93% at amino acid level), with two strains from Philippines collected in previous years (acc. n° JN415517 collected in 2010; acc. n° KR919819 collected in 2012). Moreover, the phylogenetic analysis of the strain S2014-358 from Thailand, showed the higher similarity (86% homology at nucleic acid level and 92% at amino acid level) with a Gene Bank Thai strain collected in 2010 (acc. n° JN415528). The strain S2014-358 showed the same 92% similarity at amino acid level also with other Thai strains collected in 2013 (acc. n° KJ545455 and KF887994). In Fig. 1b is reported the DENV-2 tree: 7 out of 8 strains (S2014-368, S2015-409, S2015-465, S2014-482, S2015-477, S2014-478 and S2015-512) clustered in the Cosmopolitan genotype: 4 of them (S2015-409, S2015-465, S2015-478, S2015-482), from Thailand and India, showed a high similarity with Indian isolates sequences (2001-DQ448236 and 2011-KF364514) both at nucleic acid and amino acid level (90–98% and 100% respectively). The S2014-382 sequence, from Santo Domingo, clustered in the American/Asian genotype, showing a strong homology (85 and 100% in nucleic and amino acid composition, respectively) with the strain collected in Puerto Rico in 2013 (acc. n° LN870427). The two DENV-3 strains identified in this study (S2014-339 from Cuba, and S2015-517 from unknown geographic area) clustered in genotype III, showing 93–100% of identity with the GenBank strains collected in Central American and Caribbean areas (acc. n° DQ341204 and level (Fig. 1c). The DENV-4 sequence (S2015-466, from Thailand) showed a strong homology with an isolate collected in 2013 from Myanmar area (acc. n° KJ470765) (similarity of 90 and 100% at nucleic and amino acid level, respectively), and distance values of 73–80% at nucleic acid level and 83–93% at amino acid level, with other sequences of different years from Thailand (acc. n° AY618990 collected in 1991, AY618980 collected in 1998, AY618992 collected in 2001, and EU448454 collected in 2007) (Fig. 1d). CHIKV sequences obtained from patients S2015-416 (for which the geographic area of origin of the infection was not known) and S2015-422, from Colombia, were aligned with 30 GenBank sequences with known dates and locations. From the analysis of the phylogenetic tree, the two sequences were shown to belong to the Asian genotype (Fig. 2). The S2015-416 sequence showed a 100% identity both at nucleic and amino acid level with an isolate collected in 2008 in Indonesia (acc. n° KC879577). The S2015-422 sequence showed the strongest homology with a Brazilian isolate (acc. n° KP164572) (98 and 96% similarity at nucleic and amino acid level, respectively).

**Table 8:** DENV and CHIKV sequences characterized in this study

Isolate ID	Travel location	Genotype	Year isolated	GenBank accession no.
DENV-1				
S2014-358	Thailand	I-Asian	2014	LN870423
S2014-376	Bali, Indonesia	I-Asian	2014	LN870425
S2015-510	?	I-Asian	2015	LN999960
S2015-460	French_Polynesia	I-Asian	2015	LN999954
S2015-475	Philippines	IV-South Pacific	2015	LN999955
S2015-423	Oceania	IV-South Pacific	2015	LN879497
S2014-383	Manila, Philippines	IV-South Pacific	2014	LN870426
S2015-431	Maldives	V-African/American	2015	LN879498
S2015-458	Maldives	V-African/American	2015	LN999951
S2015-481	Mexico	V-African/American	2015	LN999958
S2015-425	Haiti	V-African/American	2015	LN879499
DENV-2				
S2014-368	?	Cosmopolitan	2014	LN870428
S2015-477	Maldives	Cosmopolitan	2015	LN999956
S2015-512	?	Cosmopolitan	2015	LN999961
S2015-409	Thailand	Cosmopolitan	2015	LN999950
S2015-465	Thailand	Cosmopolitan	2015	LN999952
S2015-482	India	Cosmopolitan	2015	LN999959
S2015-478	Thailand and Cambodia	Cosmopolitan	2015	LN999957
S2014-382	Santo Domingo, Dominican Republic	America/Asian	2014	LN870427
DENV-3				
S2015-517	?	III	2015	LN999962
S2014-339	Cuba	III	2014	LN870424
DENV-4				
S2015-466	Thailand	I	2015	LN99995
CHIKV				
S2015-416	?	Asian	2015	LN879501
S2015-422	Colombia	Asian	2015	LN879500

**Table 9:** DENV and CHIKV reference sequences

Virus strain	Location	Genotype	Year isolated	GenBank accession no.
DENV-1				
NC14-17042014-4554	New Caledonia	I-Asian	2014	KM212960
China/GD-D13001	Thailand	I-Asian	2013	KJ545455
DENV-1/8/Thailand/01/2013	Thailand	I-Asian	2013	KF887994
Khabar 2759	Khabarovsk, Far East, Russia	I-Asian	2012	KJ417841
SL_2012_GS0319	Sri-Lanka	I-Asian	2012	KJ26662
MKS-WS81	Indonesia	I-Asian	2010	KC762639
D1/Vietnam/1012aTw	Viet Nam	I-Asian	2010	JF967953
Thailand 2010	Thailand	I-Asian	2010	JN415528
D1/IDN/Bali_033/2010	Indonesia	I-Asian	2010	KM216676
-	Cambodia	I-Asian	1998	AF309641
GZ/80	China	I-Asian	1980	AF350498
PUO 359	Thailand	I-Asian	1980	AF425630
16007	Thailand	II-Thailand	1964	AF180817
TH-SMAN	Thailand	II-Thailand	1954	D10513
P72-1244	Malaysia	III-sylvatic	1972	EF457905
D1/Hu/Philippines/NIID13/2016	Philippines	IV-South Pacific	2016	LC128301
FI/DB170/2014	Fiji	IV-South Pacific	2014	KM279390
Phil2012	Philippines	IV-South Pacific	2012	KR919819
Philippines 2010	Philippines	IV-South Pacific	2010	JN415517
WS01/190801-769	Samoa	IV-South Pacific	2001	JQ655095
A88	Indonesia	IV-South Pacific	1988	AB074761
AUS HCS1	Australia	IV-South Pacific	1983	AF425611
PRS 228682	Philippines	IV-South Pacific	1974	AF425627
Guangzhou/2014/4	China	V-African/American	2015	KT751343
Wenzhou-Human-1	China	V-African/American	2014	KR024708
9/D1/Del/2013	India	V-African/American	2013	KU166895
AO/DB132/2013	Angola	V-African/American	2013	KM277610
DENV-1/NI/BID-V7696/2012	Nicaragua	V-African/American	2012	KF973475
ARC-73-12	Puerto Rico	V-African/American	2012	KF444913
D1/Mexico/Ixtaczoquitlan/17/2007	Mexico	V-African/American	2007	HM171564
ThD1_0673_80	Thailand	V-African/American	1980	AY732474
715393	India	V-African/American	1971	JF297579
IBH 28328	Nigeria	V-African/American	1968	AF425625
DENV-2				
SG(EHI)D2/18944Y13	Singapore	Cosmopolitan	2013	KR779784
D2/IDN/Lombok_087/2012	Bali, Indonesia	Cosmopolitan	2012	KM216718
D2/IDN/Bali_103/2012	Bali, Indonesia	Cosmopolitan	2012	KM216731
D2/THA/086/2012	Thailand	Cosmopolitan	2012	KM216717
D2/TLS/Timor_078/2012	East Timor	Cosmopolitan	2012	KM216712
D2/IDN/Bali_108/2012	Indonesia	Cosmopolitan	2012	KM216736
12/GZ/12851	China	Cosmopolitan	2012	KF060919
D2/IDN/Bali_075/2011	Bali, Indonesia	Cosmopolitan	2011	KM216709
D2/IN/RGCB921/2011	India	Cosmopolitan	2011	KF364514
10/GZ/11864	China	Cosmopolitan	2010	JN009092
Philippines 2010b	Philippines	Cosmopolitan	2010	JN568265
D2/IDN/Jakarta_060/2010	Indonesia	Cosmopolitan	2010	KM216708
SG(EHI)D2/63481Y10	Singapore	Cosmopolitan	2010	JN030340
DENV-2/VN/BID-V735/2006	Viet Nam	Cosmopolitan	2006	EU482672
D2/SG/05K3330DK1/2005	Singapore	Cosmopolitan	2005	EU081178
GWL177 INDI-01	India	Cosmopolitan	2001	DQ448236
2784-DF-11/18/2002	Taiwan	Cosmopolitan	2002	DQ645556
MD922	Viet Nam	Asian II	2003	GU434156
40247	Brazil	Asian II	1990	L10041
ThD2_0284_90	Thailand	Asian II	1990	DQ181801
PR/DB189/2013	Puerto Rico	American/Asian	2013	KM279409
DR59/01	Dominican Republic	American/Asian	2001	AB122022
Cuba115/97	Cuba	American/Asian	1997	AY702050
IQT-1950	Perù	American	1995	DQ917242
D2/TO/UH04/1974	Tonga	American	1974	HM582117
Laos 2010	Laos	Asian I	2010	JN568244
D2/Myanmar/1007aTw	Myanmar	Asian I	2010	JF968026
D2/LAO/043/2010	Laos	Asian I	2010	KM216697
D2/Laos/1007aTw	Laos	Asian I	2010	JF968021
DENV-2/KH/BID-V2062/2007	Cambodia	Asian I	2007	GQ868624
Myan0207a/Tw	Myanmar	Asian I	2002	DQ518651
DENV-2/TH/BID-V2311/2001	Thailand	Asian I	2001	FJ744725
GD08/98	China	Asian I	1998	FJ196851
ThD2_0168_79	Bangkok, Thailand	Asian I	1979	DQ181805
DAK Ar 2022	Burkina Faso	sylvatic	1980	DQ917247
DAK Ar 2039	Burkina Faso	sylvatic	1980	DQ917246
DENV-3				
D3/IDN/Bali_007/2010	Indonesia	I	2010	KM216737
D3/Malaysia/1012bTw	Malaysia	I	2010	JF968112
H87	Philippines	I	1987	M93130
80-2	China	I	1980	AF317645
D3-73NIID	Japan	I	1973	AB111085
LN2632	Malaysia	II	1999	AF147459
D89-273	Thailand	II	1989	AY145715
PaH881/88	Thailand	II	1988	AF349753
ThD3_0046_83	Thailand	II	1983	AY676358
ThD3_0040_80	Thailand	II	1980	AY676359
ThD3_0033_74	Thailand	II	1974	AY676360
D3BR/ST14/04	Brazil	III	2004	DQ118882
-	Singapore	III	2004	AY662691
D3PY/AS12/02	Paraguay	III	2002	DQ118884
BR74886/02	Brazil	III	2002	AY679147
Cuba_167_2001	Cuba	III	2001	KT726341
Cuba580/01	Cuba	III	2001	AY702030
D3/H/IMTSSA-SRI/2000/1266	Sri Lanka	III	2000	AY099336
00-28-1HuNIID	Cambodia	III	2000	AB111081
6883/YUCATAN-MX/97	Yucatan	III	1997	DQ341204
1339	Puerto Rico	IV	1997	AY146761
Human, Tahiti 1965	Tahiti	IV	1965	L11439
DENV-4				
D4/RL196/Myanmar/2013	Myanmar	I	2013	KJ470765
D4/Thailand/0702aTw	Thailand	I	2007	EU448454
D4/Cambodia/0509aTw	Cambodia	I	2005	EU448455
ThD4_0485_01	Thailand	I	2001	AY618992
ThD4_1142_98	Thailand	I	1998	AY618980
ThD4_0348_91	Bangkok, Thailand	I	1991	AY618990
SPH317947	Brazil	II	2011	JN092553
DENV-4/VE/BID-V1156/2007	Venezuela	II	2007	GQ868645
DENV-4/CO/BID-V3412/2005	Colombia	II	2005	CQ868585
D4MY02-26658	Malaysia	II	2002	FN429922
8976/95	Singapore	II	1995	AY762085
D4/PR/M35/1985	Puerto Rico	II	1985	GU318316
ThD4_0476_97	Bangkok, Thailand	III	1997	AY618988
ThD4_0017_97	Bangkok, Thailand	III	1997	AY618989
P75-514	Malaysia	IV	1975	AF231723
P73-1120	Malaysia	IV	1973	AF231724
CHIKV				
10Mdy105	Myanmar	East Central South African	2010	KF590567
GD139	China	East Central South African	2010	HQ846358
TN06310	India	East Central South African	2010	HM159388
CU-Chik_OBF	Thailand	East Central South African	2009	GU908223
0901aTw	Malaysia	East Central South African	2009	FJ807895
0812bTw	Malaysia	East Central South African	2008	FJ807893
CU-Chik10	Thailand	East Central South African	2008	GU301780
FD080178	China	East Central South African	2008	GU199352
0810aTw	Bangladesh	East Central South African	2008	FJ807898
SGEHICHT077808	Singapore	East Central South African	2008	FJ445484
ITA07-RA1	Italy	East Central South African	2007	EU244823
DRDE-07	India	East Central South African	2007	EU372006
LR2006_OPY1	Reunion	East Central South African	2006	DQ443544
0611aTw	Singapore	East Central South African	2006	FJ807896
SL11131	Sri Lanka	East Central South African	2006	AB455493
06-027	Reunion	East Central South African	2005	AM258993
UgAg4155	Uganda	East Central South African	1982	HM045812
Vereeniging	South Africa	East Central South African	1956	HM045792
S27-African prototype	Tanzania	East Central South African	1953	AF369024
Ross low-psg	Tanzania	East Central South African	1953	HM045811
TR206/H804187	Brazil	Asian	2014	KP164572
0811aTw	Indonesia	Asian	2008	FJ807891
2008900245-BDG E1	Indonesia	Asian	2008	KC879577
MY021IMR/06/BP	Malaysia	Asian	2006	EU703762
PhH15483	Philippines	Asian	1985	HM045790
Gibbs 63-263	India	Asian	1963	HM045813
TH35	Thailand	Asian	1958	HM045810
HD 180760	Senegal	West African	2005	HM045817
37997	Senegal	West African	1983	AY726732

## Discussion

In this study, we present the results of laboratory diagnosis of imported Arbovirus infections in Italy, in the period from July 2014 to October 2015. As it is well known, dengue is endemic throughout the tropics and subtropics, and its global prevalence has grown dramatically in recent years. Indeed, we found that DENV infection was the most frequently detected imported arboviral infection among our patients. Moreover, all four known DENV genotypes were detected. An increase in imported CHIKV cases was also observed, as already documented in Spain, mainland France, and Northern Italy, along with the first identification of ZIKV imported cases; both findings are attributable to the recent dramatic spread of both CHIKV and ZIKV in previously unaffected areas [27, 42–44]. The continuous expansion of the areas with Arbovirus circulation, together with the dispersion of *Aedes* mosquitoes *spp.*, which are known or might be competent vectors [45], may increase the risk of outbreaks also in temperate climate areas. In Italy, the widespread presence of *Ae. albopictus* throughout the country, and the recent introduction and spread of new species [46, 47], make this risk particularly high. However, no autochthonous transmission chains have been recorded in our country in the period between July 2014 and October 2015.

The widespread circulation of CHIKV and ZIKV in areas until recently known to be endemic only for DENV represents a matter of concern for the potential risk of introduction in temperate regions, and raises significant diagnostic issues. In particular, problems related to the broad cross-reactivity of closely related viral agents, and the lack of well validated and standardized, commercially available tests (as it is for ZIKV), or the non-optimal performance of available tests, particularly when different viral agents co-circulate in the same areas, need to be addressed. To this regard, ZIKV imported cases are increasingly being reported, as a consequence of the continuous spread of the infection in south and central America [16, 48], leading to an increase in the requests for diagnosis. Criticisms in ZIKV diagnosis have been outlined recently [16], particularly for pregnant women [49], following the alert for the possible association between this infection and neonatal microcephaly [50].

From this scenario, the need for a careful evaluation of the diagnostic tools available for these infections clearly emerges. At this aim, in this work we have defined “our” criteria for case definition (Table 3) on the basis of the results of the diagnostic tests routinely used in our laboratory. However, it must be underlined that the final case definition for each patient is up to the clinician, and at this aim criteria are well defined in the National Plan for Arbovirus surveillance [30, 31] issued annually by the Italian Ministry of Health.

Molecular approaches for the diagnosis of viral infections are the most rapid as well as sensitive and specific. Moreover, sequencing and phylogenetic analysis of detected viruses can contribute to the knowledge of circulating viral strains and of the degree of their genetic variability. However, as suggested by our data, the use of molecular techniques is limited by the short duration of viremia during the course of the infection. With respect to serological diagnosis, we assessed some limitations in the sensitivity and/or specificity of the ELISA IgM kits routinely used in our laboratory, and also some degree of discrepancy with IgM results obtained by different laboratories. We have estimated approximately 14.5% of false positive and 11.0% false negative results for DENV, and approximately 9.6% of false negatives for CHIKV. Although the main purpose of our study was not a detailed analysis of the performances of different IgM detection systems, our data strengthen the need to confirm the diagnosis of cases defined as probable on the basis of IgM tests results. With respect to the PRNT, we have defined as “borderline” results those in which 50%,(i.e., less than 80%) of plaque reduction was observed. We found that b.l. PRNT results can be obtained in different situations. In most cases, they can be observed in samples collected soon after the onset of symptoms, and can be considered as an early, specific, response to the infection. In some cases, however, b. l. PRNT results can be due to infection by a closely related virus: we have observed a b.l. PRNT result for ZIKV in a DENV confirmed, DENV PRNT positive case; and, conversely, a DENV b.l. PRNT result in a ZIKV confirmed, ZIKV PRNT positive case. Indeed, even if PRNT is considered the most specific test, there can be some degree of cross-reactivity, thus b.l. PRNT results should always be considered cautiously. Finally, few cases showed b.l. PRNT results for several viruses (such as DENV, CHIKV and WNV), which probably represents a non-specific response of unknown origin, maybe due to an underlying, still undefined pathology.

## Conclusion

DENV infection was the most frequently diagnosed cause of illness among travelers, and all four genotypes were detected. An increase in imported CHIKV cases and the first imported ZIKV cases were detected. Major diagnostic issues highlighted in our study are sensitivity limitations of molecular tests, and the importance of PRNT to confirm serological results for the differential diagnosis of Arboviruses. Moreover, the implementation of a network of laboratories involved in surveillance activities throughout the country may greatly improve the preparedness for a rapid a proper recognition of a possible autochthonous outbreak. Finally, the continuous evaluation of laboratory findings in the context of surveillance activities can be of great importance for the development of novel diagnostics, and for field evaluation of the impact of viral diseases, also in view of vaccine development and use.

## Abbreviations

*Ae*.: *Aedes*
b.l.: Border line
CHIKV: Chikungunya virus
DENV: Dengue virus
ISS: Istituto Superiore di Sanità
NRLA: National Reference Laboratory for Arboviruses
PCR: Polymerase chain reaction
PRNT: Plaque Reduction Neutralization Test
WHO: World Health Organization
WNV: West Nile virus
ZIKV: Zika virus
