# Erratum to: Imported arboviral infections in Italy, July 2014–October 2015: a National Reference Laboratory report

**DOI:** 10.1186/s12879-017-2426-5

**Published:** 2017-05-10

**Authors:** Claudia Fortuna, Maria Elena Remoli, Caterina Rizzo, Eleonora Benedetti, Cristiano Fiorentini, Antonino Bella, Claudio Argentini, Francesca Farchi, Concetta Castilletti, Maria Rosaria Capobianchi, Lorenzo Zammarchi, Alessandro Bartoloni, Nadia Zanchetta, Maria Rita Gismondo, Luca Ceccherini Nelli, Giustina Vitale, Franco Baldelli, Pierlanfranco D’Agaro, Giuseppe Sodano, Giovanni Rezza, the Arbovirus Working Group, Giulietta Venturi

**Affiliations:** 1 Department of Infectious Diseases, Istituto Superiore di Sanità, Rome, Italy; 2 National Center for Epidemiology and Health Promotion, Istituto Superiore di Sanità, Rome, Italy; 3 Laboratory of Virology, National Institute for Infectious Diseases Lazzaro Spallanzani, IRCCS, Rome, Italy; 4 Infectious and Tropical Diseases Unit, Careggi University Hospital, Florence, Italy; 5 Department of Experimental and Clinical Medicine, University of Florence, Florence, Italy; 6 Clinical Microbiology, Virology and Bioemergency, Luigi Sacco Hospital, Milan, Italy; 7 Virology Section and Retrovirus Centre of the Department of Translational Research NSMT, University of Pisa, Pisa University Hospital (Azienda Ospedaliero-Universitaria Pisana), Pisa, Italy; 8 Department of Laboratory Diagnosis, Palermo University Hospital (Azienda Ospedaliera Universitaria Policlinico Palermo), Palermo, Italy; 9 Clinic of Infection Diseases, Department of Medicine, University of Perugia, Perugia, Italy; 10 Department of Medical, Surgical and Health Sciences, University of Trieste, Trieste, Italy; 11 Institute for Maternal and Child Health—IRCCS Burlo Garofolo, Trieste, Italy; 12 UOC Microbiology and Virology, Hospital for Infectious Diseases “D. Cotugno”, AO dei Colli (Monaldi, Cotugno, CTO), Naples, Italy

## Erratum

After the publication of this work [1] it was noticed that due to a typesetting error the corresponding author ‘Giulietta Venturi’ was omitted from the author list of the PDF. The original article has been updated to include ‘Giulietta Venturi’ as the last author.

The publisher apologises for this error.
